# Health profiles and socioeconomic characteristics of nonagenarians residing in Mugello, a rural area in Tuscany (Italy)

**DOI:** 10.1186/s12877-020-01689-3

**Published:** 2020-08-15

**Authors:** Cosmo Strozza, Patrizio Pasqualetti, Viviana Egidi, Claudia Loreti, Federica Vannetti, Claudio Macchi, for the Mugello Study Working Group, Guglielmo Bonaccorsi, Roberta Boni, Chiara Castagnoli, Francesca Cecchi, Francesca Cesari, Francesco Epifani, Roberta Frandi, Betti Giusti, Maria Luisa Eliana Luisi, Rossella Marcucci, Raffaello Molino-Lova, Anita Paperini, Lorenzo Razzolini, Francesco Sofi, Nona Turcan, Debora Valecchi, Luca Padua

**Affiliations:** 1 Interdisciplinary Centre on Population Dynamics, University of Southern Denmark, J.B. Winsløws Vej 9B, 2nd floor, 5000 Odense C, Denmark; 2 Department of Statistical Sciences, Sapienza University of Rome, Viale Regina Elena 295, 00161 Rome, Italy; 3 Fatebenefratelli Foundation for Health Research and Education, Via della Lungaretta 177, 00153 Rome, Italy; 4 Fondazione Policlinico Universitario A. Gemelli IRCCS, Largo Agostino Gemelli 8, 00136 Rome, Italy; 5 IRCCS Fondazione Don Carlo Gnocchi, Via di Scandicci 269, 50143 Florence, Italy; 6 Department of Geriatrics, Neurosciences and Orthopaedics, Università Cattolica del Sacro Cuore, Largo Francesco Vito 1, 00168 Rome, Italy; 7 UOC Neuroriabilitazione ad Alta Intensità, Fondazione Policlinico Universitario A. Gemelli IRCCS, Largo Agostino Gemelli 8, 00136 Rome, Italy

**Keywords:** Aging, Health, Health profiles, Nonagenarians, Oldest-old, Latent class analysis, Socioeconomic status, Italy

## Abstract

**Background:**

Health, as defined by the WHO, is a multidimensional concept that includes different aspects. Interest in the health conditions of the oldest-old has increased as a consequence of the phenomenon of population aging. This study investigates whether (1) it is possible to identify health profiles among the oldest-old, taking into account physical, emotional and psychological information about health, and (2) there are demographic and socioeconomic differences among the health profiles.

**Methods:**

Latent Class Analysis with covariates was applied to the Mugello Study data to identify health profiles among the 504 nonagenarians residing in the Mugello district (Tuscany, Italy) and to evaluate the association between socioeconomic characteristics and the health profiles resulting from the analysis.

**Results:**

This study highlights four groups labeled according to the posterior probability of determining a certain health characteristic: “healthy”, “physically healthy with cognitive impairment”, “unhealthy”, and “severely unhealthy”. Some demographic and socioeconomic characteristics were found to be associated with the final groups: older nonagenarians are more likely to be in worse health conditions; men are in general healthier than women; more educated individuals are less likely to be in extremely poor health conditions, while the lowest-educated are more likely to be cognitively impaired; and office or intellectual workers are less likely to be in poor health conditions than are farmers.

**Conclusions:**

Considering multiple dimensions of health to determine health profiles among the oldest-old could help to better evaluate their care needs according to their health status.

## Background

Currently, the world’s population is aging, and the number of oldest-old people is increasing considerably [1, 2]. For most developed countries, the share of nonagenarians in Italy increased by approximately 23 times in the last 70 years (from 0.06% in 1950 to 1.37% in 2020) and is expected to continue growing during the next several years, according to the World Population Prospects [3], reaching 3.27% in 2050. Consequently, a greater demand for medical care might be expected from this segment of the population. According to the Italian General State Accounting Department, people aged 65 and above had higher per capita medical expenditures in 2018 [4]. For this reason, it is becoming increasingly important to be able to appropriately measure the health of elderly adults as well as that of the oldest-old people [5] and understand which factors are related to so-called “healthy aging”. This has been performed extensively among less older people in recent decades. However, as a consequence of the increasing number of oldest-old people in Western societies and their health characteristics and needs, it is only in recent years that studies focusing on the oldest-old have been conducted, aiming to understand the potential drivers of good health conditions at extremely old ages [6–10]. These studies have always focused on a specific dimension of health, such as cognition, physical and functional status or morbidities. However, health care needs are the result of a complex system of diseases, syndromes or health characteristics that cannot be described by a single dimension of health [11–14]. To consider the multidimensionality of individual health status, it is necessary to exploit a person-centered approach that is based not on the relationships among variables but rather on the characteristics of the individuals. This approach allows people to be distinguished into groups by taking only their individual characteristics into account [11, 13].

To capture the heterogeneity of health status and evaluate the social disparities among individuals, researchers suggest the use of latent class analysis (LCA) as a person-centered approach [11–13]. LCA is a subset of structural equation modeling suitable for addressing multidimensional concepts, as in the case of health, to find groups of cases with similar characteristics in multivariate categorical data. The use of LCA in population health studies is extensive, with applications that vary from younger [15] to older individuals and elderly people [12–14, 16–24]. Some scholars used this approach to identify profiles of health by considering functional, cognitive and psychological indicators [12–14, 16, 17, 22], with some evaluating socioeconomic differences among the health profiles [12, 13, 17, 22] and others predicting the health care expenditures of people belonging to different groups [14, 16]. Other researchers have applied a person-centered approach to identify profiles within a single aspect of health, such as morbidities [15, 19, 25], physical status [21], and depression [20], by considering several outcomes of the same health dimension. According to the existing literature, LCA could be used to identify groups of individuals requiring specific forms of health care and to predict their health care needs and expenditures. This approach could also help policymakers understand which groups of people to target with their interventions. The recent COVID-19 pandemic has again highlighted, especially in Italy, how vulnerable people are, such as the oldest-old and multichronic patients, which are groups that merit greater health policy focus [26].

It is also well documented that among elderly adults, demographic and socioeconomic characteristics influence health status and, consequently, health care needs and utilization [13, 27, 28]. Fewer researchers have evaluated this relationship among extremely old people, suggesting the persistence of social disparities in health, even in the last stages of life [29]. Gender, education and income were found to be associated with different health outcomes among the oldest-old individuals, prompting further investigation in this direction [6, 29–32]. Evaluating the existence of a demographic and socioeconomic gradient in health among the oldest-old population could drive the attention of policymakers toward people who need interventions.

Despite the recognized advantage of using a person-centered approach for capturing the heterogeneity of health among elderly people, there is still not much evidence relating to health profiles among the oldest-old and the extremely-old populations [33]. To fill this gap in the literature, we analyzed data from the Mugello Study [34], which included 504 nonagenarians from a rural area in Tuscany (Italy) called Mugello. Our aim is to determine whether it is possible to classify oldest-old people according to their multidimensional health status, defined by physical, cognitive and psychological health, to help in choosing the best care needed by this growing segment of the population. Furthermore, we investigate whether there are demographic and socioeconomic differences among their health profiles, fueling the debate on social disparities in health in the last stages of life.

## Methods

### Study population and measures

The study population comes from the Mugello Study [10], which aimed to evaluate the aging process, focusing on different health aspects among nonagenarians living in 9 of the 11 municipalities of the Mugello area in Tuscany (Italy). It comprised 504 individuals representing approximately 65% of all nonagenarians living in that geographical territory in 2012. The participation rate was 69% after the exclusion of potential participants who died before being interviewed or who were not found. More information about the study design and survey methods is available in Molino-Lova et al. [10].

Much information about the individual health conditions of nonagenarians has been collected. For some of the health tests, it was not possible to assess the health status of several patients. Individuals who were not tested due to their (very) poor health conditions were categorized as nontestable. Being nontestable is considered the worst health condition for each of the variables, including this category. Variables have been categorized according to the existing literature. Cognitive function was measured according to the Mini-Mental State Examination (MMSE): the higher the score (0–30), the better the cognitive status is [35]. MMSE scores were divided into three categories to distinguish people with severe (0–17), mild (18–23), and no cognitive impairment (24–30) [36]. Functional status was assessed according to the ability to perform five of the activities of daily living (ADLs) (eating, dressing, bathing, toileting, transferring) [37]. The number of ADLs that people could manage independently was used to distinguish between the non- (0), semi- (1–4), and fully-autonomous (5) oldest-old individuals [38]. Mugello’s nonagenarians were classified as disease-free (0), single-disease (1), and comorbid (2+) according to the number of chronic diseases (cardiovascular, neurological, pulmonary, connective tissue, gastroenterological, endocrine, renal, oncological, immunodeficiency syndrome) reported. The Geriatric Depression Scale (GDS) was used to evaluate depression status: the higher the score (0–15), the higher the level of depression is [39]. GDS scores were divided into three categories to distinguish nondepressed (0–4), depressed (5–15), and nontestable individuals [40]. Self-rated health status was assessed using the Italian version of the Short Form-12 questionnaire (SF-12) from which it was possible to obtain the two synthetic indicators combining the 12 items together: the Physical and Mental Component Summaries (PCS and MCS) [41]. The PCS and MCS were divided into three categories: those who scored higher (or equal) than the average were considered to be in good health, those who scored lower than the average were considered to be in poor health, and nontestable individuals were considered to be in the worst health. It was also possible to obtain the global self-rated health (SRH) of the individual from the SF-12, according to the first item of the questionnaire (in general, you would describe your health status as…). It was divided into three categories to distinguish among nonagenarians declaring excellent/very good/good health, declaring acceptable/poor health and being nontestable.

The results are controlled for age (90–91, 92–94, 95+), gender, education (0–2, 3, 4–5, 6+ years of education), and main occupation during the working lifespan defined according to the Italian National Institute of Statistics (ISTAT) classification of jobs [42]: farmer; housewife; and low-skilled (laborer or unskilled worker) or medium-skilled (office, industry or intellectual worker) work.

### Statistical analysis

Health is a complex state involving different aspects or dimensions. To capture the heterogeneity of the health status among the oldest-old individuals, we supposed that Mugello’s nonagenarians could belong to unobserved or latent classes according to their health characteristics. For this purpose, we chose LCA, which aims to group individuals into classes according to their indicator patterns. Each class includes individuals with similar characteristics that nonetheless differ from the characteristics of those in other classes.

LCA was used to identify different health profiles according to the health condition through the variables described in the previous paragraph, controlling for demographic and socioeconomic characteristics. LCA with covariates is an extension of the basic LCA, permitting the inclusion of covariates to predict an individual’s latent class membership [43, 44].

We performed the LCA twice, including the same variables: once on the whole study population and once on the subsample of testable individuals. Since we expected to obtain in the first analysis a group populated by only nontestable individuals, we excluded those people in the second analysis to capture more heterogeneity in health status for the remaining oldest-old individuals. The effect of the covariates has been estimated with the “one-step” technique to obtain less biased coefficients: they are estimated simultaneously as part of the latent class model [45, 46].

Suppose a latent class model with *C* classes is to be estimated according to *m* categorical variables and a covariate *x*. Let Y_*i*_ = (Y_i1_, …, Y_iM_) be the vector of an individual’s response to the *M* variables, where Y_*im*_ = 1, 2, …, r_m_. Let c_i_ = 1, 2, …, C is the latent class membership of the individual to the class; let I(y = k) be the indicator function that is 1 if *y* is equal to *k* and 0 otherwise; and let λ be the probability of membership in each latent class. Then, the latent class model can be expressed as follows:

$$ \mathrm{P}\left(\mathrm{Y}=\mathrm{y}|{\mathrm{x}}_{\mathrm{i}}\right)=\sum \limits_{\mathrm{c}=1}^{\mathrm{C}}{\uplambda}_{\mathrm{c}}\left({\mathrm{x}}_{\mathrm{i}}\right)\prod \limits_{\mathrm{m}=1}^{\mathrm{M}}\prod \limits_{\mathrm{k}=1}^{{\mathrm{r}}_{\mathrm{m}}}{\uprho}_{\mathrm{m}\mathrm{k}\mid {\mathrm{c}}^{\mathrm{I}\left({\mathrm{y}}_{\mathrm{i}\mathrm{m}}=\mathrm{k}\right)}} $$

where λ_*c*_(*x*_*i*_) = *P*(*C*_*i*_ = *c*| *x*_*i*_) is a standard baseline category for the multinomial logistic model. In the case of one covariate, *λ* can be expressed as the following:

$$ {\uplambda}_{\mathrm{c}}\left({\mathrm{x}}_{\mathrm{i}}\right)=\mathrm{P}\left({\mathrm{C}}_{\mathrm{i}}=\mathrm{c}|{\mathrm{x}}_{\mathrm{i}}\right)=\frac{\exp \left\{{\upbeta}_{0\mathrm{c}}+{\mathrm{x}}_{\mathrm{i}}{\upbeta}_{1\mathrm{c}}\right\}}{1+{\sum}_{\mathrm{j}=1}^{\mathrm{C}}\exp \left\{{\upbeta}_{0\mathrm{j}}+{\mathrm{x}}_{\mathrm{i}}{\upbeta}_{1\mathrm{j}}\right\}} $$

for c = 1, …, C − 1, where *C* is the reference class in the logistic regression. As a result, the log-odds of an individual falling into latent class *c* relative to the reference class *C*, giving x_i_ as the value for the covariate, is the following:

$$ \log \left(\frac{\lambda_{c\mid c}\left({x}_i\right)}{\lambda_{C\mid c}\left({x}_i\right)}\right)={\beta}_{0c\mid c}+{\beta}_{1c\mid c}{x}_i $$

Multiple imputation was necessary to address missing values (missing at random (MAR)) to avoid a loss of precision in the analysis. The K-nearest neighbor imputation method has been used for its high performance with survey data [47]. To obtain unbiased results, neighbors are found considering all the variables available in the dataset except those that are included in the models. Five neighbors were considered to calculate the aggregated values to impute. Education, main occupation during the working lifespan, MMSE score, ADLs performed, number of chronic diseases, PCS and MCS were imputed. None had more than 7% missing values. More information about data imputation is included in Table S1 in Additional file 1.

Statistical analysis was performed using R version 3.5.0 [48], VIM [49], and the poLCA package [46].

## Results

The 504 participants included a high number of women (369); the female/male sex ratio of 2.73 confirms the higher longevity of women. The mean age ± standard deviation was 93.1 ± 3.3 in the whole study population: the men’s mean age (92.5) was lower than the women’s mean age (93.3; t-test p = 0.01). Men were more educated (64.5% of males vs 46.1% of females completed more than 3 years of school) but performed more physical jobs: 80% of males vs 52.6% of females were farmers or low-skilled workers. Overall, men had better scores on all the health measures considered in the analysis. This result is partially explained by the sex-specific age structure of the study population. Large gender differences were found in cognitive and functional status (60.7% of males vs 37.1% of females were not cognitively impaired; 61.5% of males vs 43.6% of females were autonomous). The gap in the remaining health measures is mainly due to the larger number of nontestable women (Table 1).

**Table 1:** Baseline characteristics of the nonagenarians from Mugello (2012)

Characteristics	Gender						P*
	Male		Female		Total		
	n	%	n	%	n	%	
*Study population*	135	26.8	369	73.2	504	100	
*Age (m. sd)*	92.5	2.8	93.3	3.4	93.1	3.3	< 0.001
*Education (years)*							
0-2	16	11.9	49	13.3	65	12.9	< 0.001
3	32	23.7	150	40.7	182	36.1	
4-5	63	46.7	142	38.5	205	40.7	
6+	24	17.8	28	7.6	52	10.3	
*Work (level#)*							
farmer	88	65.2	163	44.2	251	49.8	< 0.001
housewife	0	0.0	95	25.7	95	18.8	
low	20	14.8	31	8.4	51	10.1	
middle	27	20.0	80	21.7	107	21.2	
*Self-rated health*							
excellent/very good/good	84	62.2	191	51.8	275	54.6	< 0.001
acceptable/poor	34	25.2	85	23.0	119	23.6	
not testable	17	12.6	93	25.2	110	21.8	
*Mini-Mental State Examination*							
24-30	82	60.7	137	37.1	219	43.5	< 0.001
18-23	24	17.8	75	20.3	99	19.6	
0-17	29	21.5	157	42.5	186	36.9	
*Activities of Daily Living*							
5	83	61.5	161	43.6	244	48.4	< 0.001
4-1	44	32.6	158	42.8	202	40.1	
0	8	5.9	50	13.6	58	11.5	
Geriatric Depression Scale							
< 5	77	57.0	141	38.2	218	43.3	< 0.001
≥ 5	40	29.6	130	35.2	170	33.7	
not testable	18	13.3	98	26.6	116	23.0	
*Physical Component Summary*							
≥ average	75	55.6	130	35.2	205	40.7	< 0.001
< average	43	31.9	146	39.6	189	37.5	
not testable	17	12.6	93	25.2	110	21.8	
*Mental Component Summary*							
≥ average	66	48.9	136	36.9	202	40.1	0.005
< average	52	38.5	140	37.9	192	38.1	
not testable	17	12.6	93	25.2	110	21.8	
*Chronic diseases (number)*							
0	17	12.6	25	6.8	42	8.3	0.112
1	31	23.0	90	24.4	121	24.0	
2+	87	64.4	254	68.8	341	67.7	

*Male vs Female from Pearson χ2 test or t-test as appropriate

#low: laborer or unskilled worker; medium: office, industry or intellectual worker

Three latent classes were found when both the whole study population and the subsample of testable individuals were considered. This number was chosen according to the “meaning” of the classes, together with the Akaike Information Criterion (AIC) and the Bayesian Information Criterion (BIC), whose values are shown in Table 2. Every latent class has been labeled according to the posterior probabilities (*λ*) of finding a certain characteristic in the class, as shown in Table 3.

**Table 2:** Model fit statistics for 2- to 6-class models

N. classes	2	3	4	5	6
*Whole study population (n = 504)*					
AIC	5212.18	**4861.80**	7174.00	7145.64	7229.17
BIC	5372.64	**5123.59**	7537.14	7610.12	7794.99
*Testable subsample (n = 385)*					
AIC	3696.26	**3652.69**	3627.05	4113.39	4168.96
BIC	3814.86	**3850.35**	3903.77	4469.18	4603.81

Note: *AIC* Akaike Information Criterion, *BIC* Bayesian Information Criterion

**Table 3:** Health status indicator probabilities (λ) per health status profile resulting from the two LCAs

Variable	Item	Whole study population (*n* = 504)			Testable subsample (*n* = 385)		
		Latent class			Latent class		
		1	2	3	1	2	3
n	(%)	215(42.9%)	110 (21.8%)	179 (35.3%)	202 (53%)	128 (33.3%)	55 (13.7%)
Activities of Daily Living	autonomous	0.89	0.09	0.22	0.88	0.23	0.38
	semiautonomous	0.11	0.47	0.72	0.12	0.70	0.60
	not autonomous	0.00	0.44	0.06	0.00	0.07	0.02
Geriatric Depression Scale	not depressed	0.81	0.03	0.23	0.82	0.20	0.43
	depressed	0.18	0.00	0.74	0.18	0.80	0.57
	not testable	0.01	0.97	0.04			
Mental Component Summary	≥ average	0.65	0.00	0.34	0.67	0.35	0.26
	< average	0.35	0.00	0.66	0.33	0.65	0.74
	not testable	0.00	1.00	0.00			
Mini-Mental State Exam.	24-30	0.78	0.01	0.28	0.83	0.32	0.09
	18-23	0.18	0.02	0.32	0.15	0.33	0.43
	0-17	0.04	0.97	0.40	0.02	0.35	0.48
Number of chronic diseases	0	0.11	0.13	0.02	0.10	0.02	0.04
	1	0.27	0.23	0.21	0.27	0.23	0.19
	2+	0.62	0.65	0.77	0.63	0.75	0.77
Physical Component Summary	≥ average	0.73	0.00	0.26	0.71	0.09	0.88
	< average	0.27	0.00	0.74	0.29	0.91	0.12
	not testable	0.00	1.00	0.00			
Self-Rated Health	excellent/very good/good	0.92	0.00	0.43	0.91	0.26	1.00
	acceptable/poor	0.08	0.00	0.57	0.09	0.74	0.00
	not testable	0.00	1.00	0.00			

Empty items are due to the subsampling: not testable individuals are not included in the second analysis

For both analysis 1: “healthy group”; respectively 2: “Severely unhealthy group” and “Unhealthy group”; and respectively 3: “Partially satisfied unhealthy group” and “Physically healthy with cognitive impairment group”

LCA performed on the whole study population resulted in three health profiles. The first class is characterized by a high probability of being autonomous (*λ* = 0.89), not depressed (*λ* = 0.81), not cognitively impaired (*λ* = 0.78), perceiving good SRH (*λ* = 0.92), and having values of PCS and MCS higher than or equal to the average (respectively, *λ* = 0.73 and 0.65). This class, labeled the “healthy group”, includes 215 individuals (42.9% of the whole study population). The second class is characterized by a high probability of being semi−/not autonomous (respectively, *λ* = 0.47 and 0.44), cognitively impaired (*λ* = 0.97), and not testable for depression (*λ* = 0.97) and SRH (*λ* = 1); consequently, PCS and MCS were not testable (*λ* = 1 for both indicators). This class has been labeled the “severely unhealthy group”. It includes 110 individuals (21.8% of the whole study population), which encompassed almost all nontestable nonagenarians according to the scales in analysis that included this category (SRH, depression, PCS and MCS). The third class includes nonagenarians with a high probability of being semiautonomous (*λ* = 0.72), mild/severely cognitively impaired (respectively, *λ* = 0.32 and 0.40), depressed (*λ* = 0.74), and having PCS and MCS scores lower than the average (respectively, *λ* = 0.74 and 0.66). Despite how they performed in the objective health measures, they frequently declare a better health status: *λ* = 0.43 for declaring good SRH conditions is relatively high (poor SRH: *λ* = 0.57). For this reason, the last class, composed of 179 (35.3%) individuals, has been labeled the “partially satisfied unhealthy group”.

LCA performed on the subsample of testable individuals also resulted in three health profiles. The first class is characterized by a high probability of being autonomous (*λ* = 0.88), not depressed (*λ* = 0.82), not cognitively impaired (*λ* = 0.83), reporting good SRH (*λ* = 0.91), with PCS and MCS scores higher than or equal to the average (respectively *λ* = 0.71 and 0.67). This class has been labeled the “healthy group”. It includes 202 individuals (53% of the testable subsample) who were almost the same individuals populating the “healthy group” resulting from the first analysis. The second class is characterized by a high probability of being semiautonomous (*λ* = 0.7), depressed (*λ* = 0.81), and reporting poor SRH (*λ* = 0.74), with PCS and MCS scores lower than the average (respectively *λ* = 0.91 and 0.65). This group of 128 individuals (33.3% of the testable subsample) has been labeled the “unhealthy group”. The third group is characterized by a high probability of reporting good SRH (*λ* = 1) and being semiautonomous (*λ* = 0.60), mild/severe cognitive impairment (respectively *λ* = 0.43 and 0.48), with MCS scores lower (*λ* = 0.74) but PCS scores higher than or equal to the average (*λ* = 0.88). Posterior probabilities for depression are similar: *λ* = 0.43 not-depressed vs *λ* = 0.57 depressed. This group was labeled “physically healthy with cognitive impairment”. It included 55 nonagenarians (13.7% of the testable subsample). All the posterior probabilities are reported in Table 3.

**Fig. 1:**
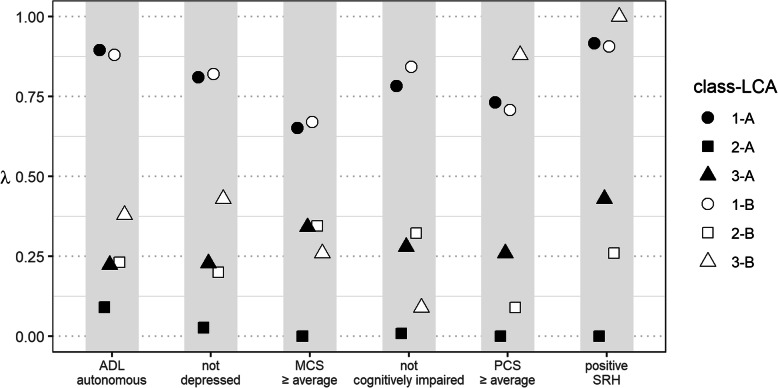
(Good) health status item probabilities (λ) per health status resulting from the two latent class analyses (LCAs). Note 1: Class 1: “Healthy group”, for both first (**a**) and second (**b**) LCAs; Class 2 for LCA-A: “Severely unhealthy group”, for LCA-B: “Unhealthy group”; Class 3 for LCA-A: “Partially satisfied unhealthy group”, for LCA-B: “Physically healthy with cognitive impairment group”. Note 2: ADLs: Activities of Daily Living; MCS: Mental Component Summary; PCS: Physical Component Summary; Positive self-rated health: excellent/very good/good self-rated health

The first class has been labeled the “healthy group” in both analyses: posterior probabilities followed a similar pattern, especially in terms of (good) health status items, as shown by the black and white circles in Fig. 1. The second class of the analysis on the whole study population was named the “severely unhealthy group” (see black squares in Fig. 1). It was composed of almost all the nontestable nonagenarians: individuals in the worst health conditions. Excluding the nontestables for the second analysis, many individuals populating the third class moved to the second, resulting in an “unhealthy group” with less *extreme* health characteristics. The consequence of this exclusion was more evident for the last (third) class obtained in both analyses. When considering all nonagenarians, we obtained the “partially satisfied unhealthy group”, i.e., people mainly in poor health conditions but not always declaring poor SRH. When excluding the nontestable nonagenarians, some of the individuals populating the third group obtained in the previous analysis moved to the second group in the second analysis. As shown in Fig. 1, the “partially satisfied unhealthy group” (first analysis) and the “unhealthy group” (second analysis) had similar posterior probabilities for the (good) health status indicators, especially in terms of functional and cognitive status. Within the second analysis, 55 out of the 385 nonagenarians composing the “physically healthy with cognitive impairment group” had a higher probability of declaring good SRH and obtaining a high PCS score than the “healthy group”, but they had poor cognitive health, sometimes had depression and were mainly semiautonomous nonagenarians.

The results are controlled for age, gender, education, and main occupation during the working lifespan (Table 4). In the analysis on the whole of Mugello’s nonagenarians, older individuals and housewives are more likely to be part of the “severely unhealthy group” instead of the “healthy group” (92–94 vs 90–91: odds ratio (OR) = 2.69; 95+ vs 90–91: OR = 7.25; housewives vs farmers: OR = 2.19), while being more educated reduces these odds (4–5 vs 3 years of education: OR = 0.49; 5+ vs 3: OR = 0.08). Being older also increases the odds of being in the “partially satisfied unhealthy group” instead of the “healthy group” (95+ vs 90–91: OR = 5.1), while both being male and a middle-level (qualified office) worker reduces it (male vs female: OR = 0.40; middle-level worker vs farmer: OR = 0.43).

**Table 4:** Odds ratios of demographic and socio-economic characteristics for the health profiles

LCA		Whole sample (*n* = 504)					Testable subsample (*n* = 385)				
Variable	Item	Coefficient	OR	Std. error	t value	Pr (>|t|)	Coefficient	OR	Std. error	t value	Pr (>|t|)
		***2*** **vs** ***1***					***2*** **vs** ***1***				
	(Intercept)	−0.88	0.41	0.33	−2.64	0.01	−0.29	0.75	0.33	−0.87	0.38
Age class (ref. 90-91)	92-94	0.99	2.69	0.34	2.92	0.00	0.10	1.11	0.38	0.27	0.79
	95+	1.98	7.25	0.37	5.38	0.00	1.68	5.37	0.37	4.55	0.00
Sex (ref. female)	male	−0.85	0.43	0.38	−2.24	0.03	−0.82	0.44	0.35	−2.32	0.02
Education (ref. 3 years)	0-2	−0.60	0.55	0.46	−1.31	0.19	0.49	1.64	0.52	0.94	0.35
	4-5	−0.72	0.49	0.32	−2.24	0.03	− 0.31	0.74	0.34	−0.89	0.37
	6+	−2.59	0.08	0.98	−2.65	0.01	−0.99	0.37	0.74	−1.33	0.19
Work (ref. farmer)	housewife	0.78	2.19	0.39	2.00	0.05	−0.20	0.82	0.50	−0.41	0.69
	low level	0.05	1.05	0.50	0.10	0.92	0.08	1.08	0.44	0.17	0.87
	middle level	−0.09	0.92	0.40	− 0.22	0.83	− 0.79	0.45	0.42	−1.89	0.06
		***3*** **vs** ***1***					***3*** **vs** ***1***				
	(Intercept)	−0.14	0.87	0.31	−0.45	0.65	−2.44	0.09	0.70	−3.51	0.00
Age class (ref. 90-91)	92-94	0.21	1.24	0.33	0.66	0.51	1.53	4.62	0.66	2.32	0.02
	95+	1.63	5.10	0.34	4.83	0.00	2.20	9.03	0.68	3.25	0.00
Sex (ref. female)	male	−0.92	0.40	0.32	−2.88	0.00	−0.50	0.61	0.73	−0.68	0.49
Education (ref. 3 years)	0-2	0.30	1.35	0.42	0.71	0.48	2.08	8.02	0.74	2.82	0.01
	4-5	−0.24	0.79	0.31	−0.77	0.44	−0.65	0.52	0.63	−1.03	0.30
	6+	−0.46	0.63	0.54	−0.85	0.40	0.89	2.43	1.06	0.84	0.40
Work (ref. farmer)	housewife	0.11	1.12	0.40	0.29	0.78	0.97	2.65	0.63	1.54	0.12
	low level	0.06	1.06	0.42	0.14	0.89	−0.80	0.45	1.09	−0.73	0.47
	middle level	−0.85	0.43	0.38	−2.26	0.02	−1.15	0.32	0.94	−1.22	0.23

For both analysis 1: “healthy group”; 2; respectively 2: “Severely unhealthy group” and “Unhealthy group”; and respectively 3: “Partially satisfied unhealthy group” and “Physically healthy with cognitive impairment group”

In the analysis on the subsample of testable individuals, as for the last class of the previous analysis, being older increases the odds of being in the “unhealthy group” instead of the “healthy group” (95+ vs 90–91: OR = 5.37), while both being male and a middle-level (qualified office) worker reduces it (male vs female: OR = 0.44; middle-level work vs farmer: OR = 0.45). Finally, being both older and less educated increases the odds of being in the “physically healthy with cognitive impairment group” instead of in the “healthy group” (92–94 vs 90–91: OR = 4.62; 95+ vs 90–91: OR = 9.03; 0–2 vs 3 years of education: OR = 8.02).

## Discussion

To identify health profiles among nonagenarians from Mugello (Tuscany - Italy), LCA was performed twice: first on the whole study population and then on the subsample of testable individuals, with nonagenarians in the “extreme” (worst) conditions having been excluded from the analysis. Removing these individuals from the analysis allowed us to capture more heterogeneity of health among the remaining oldest-old, especially among those with poor health that were hidden by the nontestable individuals.

In both analyses, three classes were identified, resulting in a total of four different health profiles within the two LCAs performed, each labeled according to the posterior probabilities of finding certain health characteristics in them. Other researchers who looked at health profiles among elderly people by considering their physical, cognitive and psychological status found two to six classes [11–13, 17, 22]. In particular, other researchers could distinguish between a larger number of classes (four to six) [11, 13, 17, 22], except for Ng et al. (2014), who identified only two profiles [12]. The fact that we found four health profiles within the two analyses means that, even at extremely old ages, there is still heterogeneity in the health conditions of the individuals. LCA allowed us to take into account the multidimensionality of health by including several health measures in the analysis. Having a larger study population could have helped to find the four profiles within a single LCA.

The “healthy group” (a), identified in both analyses and composed of almost the same individuals, and the “unhealthy group” (c), resulting from the second analysis, are consistent with other scholars’ findings among younger adults, including information on sensory health and specific chronic diseases [11, 16] or quality of life and wellbeing [17]. Additionally, among nonagenarians, it was possible to find the two extreme groups of people in overall good and poor health. The “severely unhealthy group” (b), resulting from the first analysis, confirms that nontestable individuals are a stand-alone group of people who, because of their extremely bad health conditions, cannot be tested on their health status. The “physically healthy with cognitive impairment group” (d), i.e., individuals with good self-rated health and physical condition but bad cognitive status, is similar to what Lafortune et al. (2009) called the “cognitively impaired group” in their paper on the Canadian elderly, where the authors did not include information on the perception of health [11]. However, this result is at odds with what Zammith and colleagues found in 2012, in terms of self-perceived health, among the Lothian Birth Cohort 1936 “good fitness/low spirit group” [11, 17]. It is known that one of the factors influencing the assessment of health among Italian elderly people is their physical status [50]. It is possible that, even at extremely old ages, physical health plays an important role in the self-assessment of health status. However, this could also be the result of the poor cognitive status of individuals populating the “physically healthy with cognitive impairment group”.

Certain demographic and socioeconomic characteristics were found to be associated with being part of some of the latent classes found. In this study, it is not possible to evaluate the health deterioration itself, but even at extremely old ages, being older results in having a higher probability of being in worse health. This suggests the need for further investigation on the health deterioration process among the oldest-old as it is commonly performed on the younger-old [51–53]. Males have a lower probability of being in worse general health conditions, confirming the so-called “gender paradox” also exists among the oldest-old: men are healthier than women at older ages [6, 29, 31, 54]. The level of education is known to be associated with cognitive health in later life. Researchers analyzing English and Finnish nonagenarians show how this relationship still persists at extremely old ages [29, 32, 55]. In the present study, more educated nonagenarians are less likely to belong to an “unhealthy group”, while being less educated increases the probability of being among the cognitively impaired. These results are similar to those found in younger-elderly profiles [12, 13]. Working experience is also associated with health conditions, showing different results. In line with the existing literature, a person who was a nonmanual (office) worker had a lower probability of being in bad health condition at older ages compared to someone who worked as a farmer [56, 57]. Housewives were more likely to be in the worst health conditions, similar to study findings among Finnish nonagenarians [29].

This study has public policy implications that need to be noted. Even among nonagenarians, individuals are heterogeneous in terms of health. To capture this heterogeneity by taking into account several dimensions of health, it is necessary to apply a suitable methodology. LCA has been widely used for this purpose, and policy makers should take advantage of it to identify heterogeneous groups of individuals to target with their interventions [11–14]. Analyzing different health dimensions at the same time allowed us to distinguish between the most vulnerable individuals with several health problems and those individuals with dimension-specific health deficits. According to our results, it is likely that people with poor physical health also have cognitive impairment, resulting in complex care needs. However, cognitively deteriorated individuals may be in good physical and functional status, requiring a different (specific) type of health assistance. Furthermore, health profiles were associated with socioeconomic status, showing that even among the oldest-old, the well-known socioeconomic gradient of health persists. As pointed out by Ng et al. (2014), this should suggest policy makers drive their interventions to the less advantaged groups of the population [12]. Other researchers evaluated the health care needs and expenditures among Taiwanese elderly people [14, 16], showing how they differ among the health profiles that they identified. Being able to distinguish between groups of people with different health care needs is extremely important for reducing the excess of health expenditure that may result from not considering it holistically [11].

This study has limitations that need to be noted. It is based on a cross-sectional dataset: health characteristics have been collected only once. For this reason, we were not allowed to study the causal relationship between sociodemographic characteristics and health status and profiles. Furthermore, much of the information about health status is self-reported, and cutoff points - chosen according to the existing literature - did not equate to a clinical diagnosis. Thus, it would be useful to verify their veracity with objective measures. Finally, it is important to remark that Mugello’s nonagenarians are a selected group of individuals in terms of health and mortality. Living in a rural area and following a Mediterranean diet is, for instance, something that affects this selection.

## Conclusions

Large samples of nonagenarians, for which much information has been collected about their health status, are still rare to find. Considering health as a multidimensional concept by identifying health profiles could help to better evaluate the care needs according to the different health profiles of each person, even among extremely old individuals [16, 58]. The demographic and socioeconomic gradient of health resulting from the analysis suggests that policy makers focus their interventions on specific groups of individuals at younger ages to prevent an excess of health care expenditure later on.

## Supplementary information

**Additional file 1: Table S1.** Marginal distribution pre- and post-missing values imputation of characteristics of the study population. Absolute values, percentages and differences.

## Data Availability

The data that support the findings of this study are available from *Mugello Study* but restrictions apply to the availability of these data, which were used under license for the current study, and so are not publicly available. Data are however available from the authors upon reasonable request and with permission of *Mugello Study*.

## Abbreviations

ADL: Activities of Daily Living
AIC: Akaike’s Information Criterion
BIC: Bayesian Information Criterion
GDS: Geriatric Depression Scale
ISTAT: Italian National Institute of Statistics
LCA: Latent Class Analysis
MCS: Mental Component Summary
MMSE: Mini-Mental State Examination
OR: Odds Ratio
PCS: Physical Component Summary
SF-12: Short-Form 12
SRH: Self-Rated Health
WHO: World Health Organization
