# Supplementary material for: Health profiles and socioeconomic characteristics of nonagenarians residing in Mugello, a rural area in Tuscany (Italy)
Source: BMC Geriatr. 2020 Aug 15;20:289. doi: 10.1186/s12877-020-01689-3 (PMC7429096; doi:10.1186/s12877-020-01689-3)
Supplement: Supplementary file 1 — Additional file 1: Table S1. Marginal distribution pre- and post-missing values imputation of characteristics of the study population. Absolute values, percentages and differences. [file 12877_2020_1689_MOESM1_ESM.docx]

| **Table 1: Marginal distribution pre- and post-missing values imputation of characteristics of the study population. Absolute values, percentages and differences** | | | | | |
| --- | --- | --- | --- | --- | --- |
| Characteristics | Pre-imputation | | Post-imputation | | Difference (%) |
|  | n | % | n | % |  |
| *Education (years)* |  |  |  |  |  |
| 0 2 | 65 | 13.5 | 65 | 12.9 | -0.6 |
| 3 | 166 | 34.5 | 182 | 36.1 | 1.6 |
| 4 5 | 198 | 41.2 | 205 | 40.7 | -0.5 |
| 6+ | 52 | 10.8 | 52 | 10.3 | -0.5 |
| total | 481 | 100 | 504 | 100 |  |
| *Work (level*)* |  |  |  |  |  |
| farmer | 245 | 49.4 | 251 | 49.8 | 0.4 |
| housewife | 93 | 18.8 | 95 | 18.8 | 0.1 |
| low | 51 | 10.3 | 51 | 10.1 | -0.2 |
| middle | 107 | 21.6 | 107 | 21.2 | -0.3 |
| total | 496 | 100 | 504 | 100 |  |
| *Mini-Mental State Examination* |  |  |  |  |  |
| 24 30 | 213 | 43.8 | 219 | 43.5 | -0.4 |
| 18 23 | 95 | 19.5 | 99 | 19.6 | 0.1 |
| 0 17 | 178 | 36.6 | 186 | 36.9 | 0.3 |
| total | 486 | 100 | 504 | 100 |  |
| *Activities of Daily Living* |  |  |  |  |  |
| 5 | 235 | 47.6 | 244 | 48.4 | 0.8 |
| 4 1 | 201 | 40.7 | 202 | 40.1 | -0.6 |
| 0 | 58 | 11.7 | 58 | 11.5 | -0.2 |
| total | 494 | 100 | 504 | 100 |  |
| *Physical Component Summary* |  |  |  |  |  |
| ≥ average | 187 | 39.1 | 205 | 40.7 | 1.6 |
| < average | 181 | 37.9 | 189 | 37.5 | -0.4 |
| non-testable | 110 | 23.0 | 110 | 21.8 | -1.2 |
| total | 478 | 100 | 504 | 100 |  |
| *Mental Component Summary* |  |  |  |  |  |
| ≥ average | 182 | 38.1 | 202 | 40.1 | 2.0 |
| < average | 186 | 38.9 | 192 | 38.1 | -0.8 |
| non-testable | 110 | 23.0 | 110 | 21.8 | -1.2 |
| total | 478 | 100 | 504 | 100 |  |
| *Chronic diseases (number)* |  |  |  |  |  |
| 0 | 42 | 9.0 | 42 | 8.3 | -0.7 |
| 1 | 111 | 23.9 | 121 | 24.0 | 0.1 |
| 2+ | 312 | 67.1 | 341 | 67.7 | 0.6 |
| total | 465 | 100 | 504 | 100 |  |
| *low: laborer or unskilled worker; medium: office, industry or intellectual worker | | | | | |
|  |  |  |  |  |  |
